# Disentangling the Effect of Key Parameters in Hydrogen Evolution for Rational Design of Metal–Semiconductor Photocatalysts via Self-Assembly

**DOI:** 10.1021/acsami.5c14789

**Published:** 2025-08-14

**Authors:** Chunchun Li, Ziwei Ye, Shan Xu, Nathan Skillen, Yingrui Zhang, Zehong Xu, Colby Chang, Jinlong Zhang, Peter K. J. Robertson, Steven. E. J. Bell, Yikai Xu

**Affiliations:** a School of Materials Science and Engineering, 47860 East China University of Science and Technology, Shanghai 200237, P. R. China; b Key Laboratory for Advanced Materials and Feringa Nobel Prize Scientist Joint Research Center, Frontiers Science Center for Materiobiology and Dynamic Chemistry, School of Chemistry and Molecular Engineering, 47860 East China University of Science and Technology, 130 Meilong Road, Shanghai 200237, P. R. China; c School of Chemistry and Chemical Engineering, Queen’s University Belfast, University Road, Belfast BT9 5AG, Northern Ireland, U.K.

**Keywords:** interface self-assembly, surface-accessible, nanocomposite, emulsion, photocatalyst, microcapsule

## Abstract

Fabrication of high-performance metal–semiconductor photocatalysts is a challenging problem in nanoengineering since it requires development of methods, which create strong metal–semiconductor contacts and accessible catalytic surfaces while simultaneously allowing control of the physical properties of the metal nanoparticle cocatalysts. Here, we introduce a convenient self-assembly approach for preparing highly active metal-TiO_2_ photocatalysts, which meets all these requirements. More specifically, preformed Au/Pt and TiO_2_ nanoparticles were used to generate Pickering emulsions, which were converted in situ into polymer microbeads covered in a mixed surface layer of tightly packed metal and TiO_2_ nanoparticles with photocatalytic properties. A key benefit of our synthetic approach is that it allowed the physical parameters of the photocatalyst to be controlled independently. This made the materials an ideal model system to investigate structure–property relationships in photocatalysis, which allowed us to rationalize the effect of metal size, loading, surface chemistry, and composition on hydrogen evolution efficiency. Understanding the interplay of these factors allowed the creation of photocatalysts to move away from trial-and-error and enabled us to rationally design and prepare composite photocatalysts with exceptional activity. More broadly, our self-assembly approach can be readily extended to the creation of other metal–semiconductor systems, which will pave the way for both fundamental and applied photocatalytic studies.

## Graphical abstract

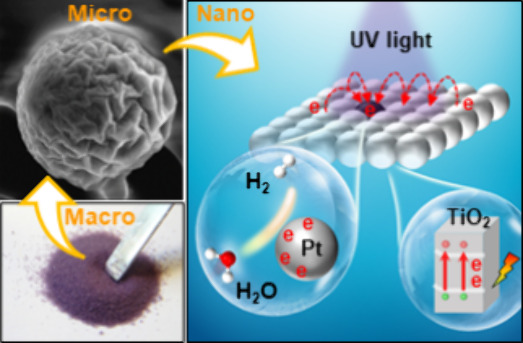

## Introduction

Hybrid nanomaterials have attracted wide research interest because they allow the creation of materials with synergistic properties that arise from the interaction between the constituent nanoparticles.^1–5^ A well-known example of such synergy is metal-TiO_2_ nanocomposites, in which the presence of metal nanoparticles significantly improves the photocatalytic properties of TiO_2_ leading to significantly enhanced performance in important potential applications including sunlight-driven water remediation and H_2_ production.^6–8^ In general, it has been shown that metal nanoparticles contribute to photocatalytic activity either by functioning as cocatalysts for electron trapping or as nanoantennae for enhancing light harvesting and/or absorption via localized surface plasmon resonance (LSPR). Also well established is the fact that the exact role of the metal nanoparticles in photocatalysts is highly complex and that the activity of the catalyst is significantly and simultaneously affected by the size, shape, loading and surface chemistry of the metal nanoparticles.^9–11^ As a result, the ability to carry out mechanistic studies, where the numerous parameters listed above are controlled in a simple and systematic fashion, to allow their impact on photocatalytic efficiency to be assessed independently, is essential for the rational design of photocatalysts with optimal functionalities.^12–15^ However, this cannot be achieved with current methods for creating composite photocatalysts, where multiple material properties are typically intertwined. For example, in conventional methods for creating metal–semiconductor photocatalysts, such as photodeposition, chemical deposition, impregnation, and physical vapor deposition, where metal nanoparticles are formed on semiconductor materials in situ, increased metal nanoparticle loading is inevitably accompanied by an increase in the size of the nanoparticles and changes to particle morphology.^16–18^

An effective way to mitigate the issue above is to take advantage of the recent developments in colloidal synthesis by using preformed metal nanoparticles for the creation of metal–semiconductor photocatalysts.^19,20^ However, bringing preformed metal and semiconductor nanoparticles together to form well-defined structures typically requires the use of organic linkers, which remain strongly adsorbed on the surface of the composite materials and are often very difficult to remove.^21–23^ This limits the surface activity of the photocatalysts and makes it challenging to tune the surface chemistry of nanoparticles to study its influence on the photocatalytic activity. As a result, although much effort has been devoted to exploring the role of metal nanoparticles in photocatalysis, there is still a lack of understanding on how the material characteristics of metal nanoparticles affect photocatalytic activity, which has meant that the construction of metal–semiconductor photocatalysts has had to rely largely on trial-and-error.

Interfacial self-assembly has been demonstrated to be a powerful and versatile approach for creating composite materials from colloidal nanoparticles,^24,25^ but its application in the creation of photocatalysts for hydrogen evolution remains unexplored. In this work, we present a fully customizable interfacial self-assembly approach for creating metal–semiconductor composite powder photocatalysts. Our method utilizes the water–oil interface as a soft-template to assemble preformed colloidal metal and semiconductor nanoparticles into Pickering emulsions, which can then be transformed into powder photocatalysts (termed nanomicro particles, NMPs) via in situ polymer precipitation. Importantly, we show that using this approach, the composition, size, morphology, loading, and surface chemistry of the metal nanoparticles in the metal–semiconductor photocatalysts can be independently and rationally controlled, which allowed us to systematically study the effect of each of these parameters on the functionality of the photocatalyst in hydrogen evolution. Our studies revealed that the optimal particle loading, i.e. particle density, varied by several order of magnitudes for metal nanoparticles with varying diameters. This unexpected phenomenon can be rationalized by considering the capture angle of photoinduced electrons by the nanoparticles, which varies depending on the particle size and the distance the electron travels before reaching the metal. Moreover, we show that these newly obtained chemical insights allowed rational design of Au/Pt-TiO_2_ NMPs using standard Au/Pt colloids and P25 TiO_2_ to achieve photocatalytic activity that outperformed state-of-the-art composite materials in hydrogen evolution. More broadly, the self-assembly approach and mechanistic insights shown in this work can be used to guide the synthesis of high-performance multifunctional nanocomposites with applications beyond photocatalysis.

## Results and Discussion

### One-Pot Preparation of NMPs

In practice, the procedure for producing NMPs was extremely straightforward since it simply involved shaking and then drying a solution mixture containing metal and metal oxide nanoparticles, polystyrene/dichloromethane solution, and tetrabutylammonium nitrate (TBA^+^NO_3_^–^) promoter. This led to the formation of NMPs, which could be stored as powder materials and redispersed in solvents in applications. Importantly, as discussed below, the physical properties of the NMPs including the average size of the NMP particle as well as the composition, surface chemistry, and morphology of the nanoparticle layer were fully customizable via simple adjustments to the synthetic procedure, which made the NMPs a versatile platform for photocatalytic research.

The successful creation of NMPs was a result of careful design, where numerous aspects of self-assembly were optimized. More specifically, as shown in Figure 1, the preparation of NMPs involved two main processes, (i) the interfacial assembly of nanoparticles to form oil-in-water Pickering emulsions and (ii) the conversion of Pickering emulsions into NMPs through in situ polymer deposition. The self-assembly of solid nanoparticles at the interface of two immiscible fluids in Step 1 is an energetically favorable process that lowers interfacial energy.^26,27^ However, presynthesized colloidal nanoparticles, such as the Au and TiO_2_ nanoparticles used in this work (see Figures S1–S3 for characterizations of the colloidal nanoparticles), typically carried strong surface charge.^28,29^ As a result, nanoparticles were prevented from assembling into densely packed structures at the water–oil interface due to strong interparticle electrostatic repulsion. This problem was mitigated by using TBA^+^NO_3_^–^ as “promoters” for self-assembly.^27^ TBA^+^ is an amphiphilic ion that carries an opposite charge from the colloidal nanoparticles. As shown in the inset of Figure 1A, during the self-assembly process, the TBA^+^ promoters sit between adjacent nanoparticles at the water–oil interface to provide charge screening without needing to adsorb onto the surface of the nanoparticles. This allowed nanoparticles to overcome interparticle electrostatic repulsion and pack densely at the water–oil interface.

**Figure 1:**
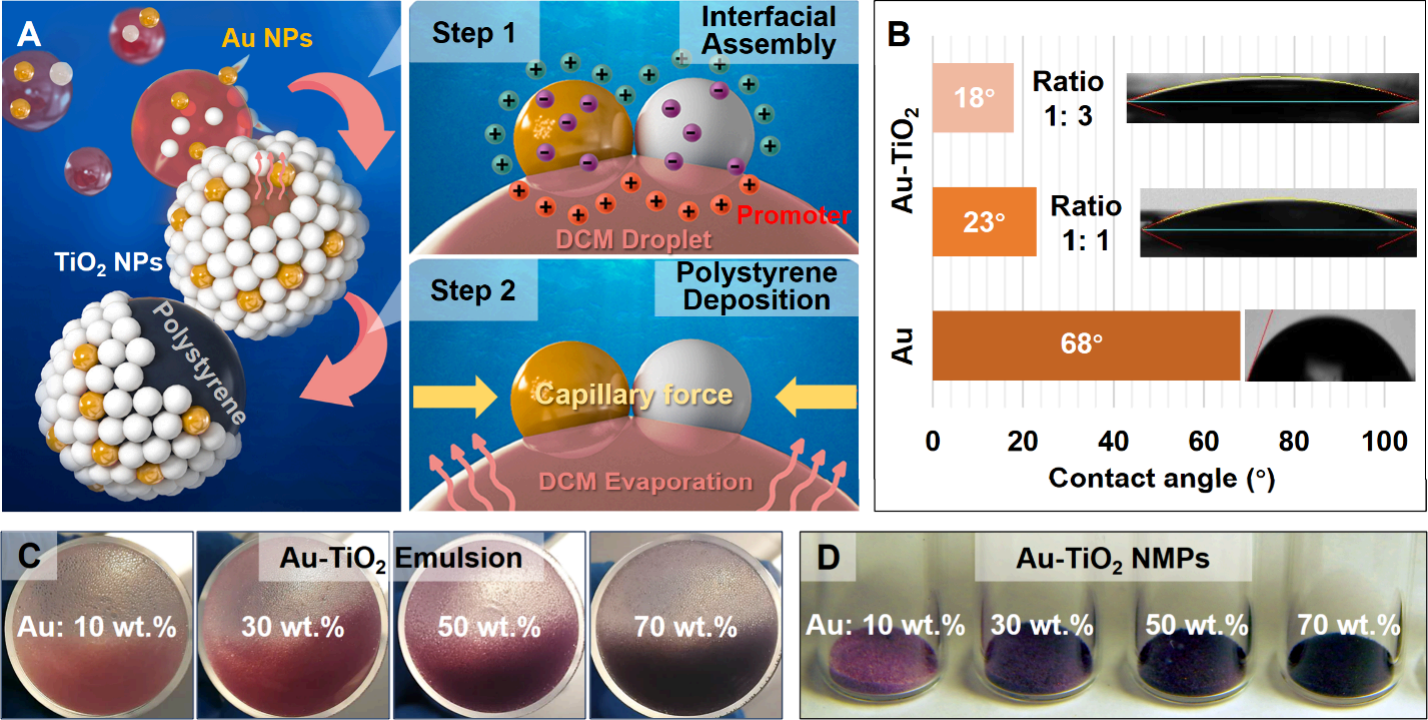
(A) Schematic illustration of the formation of metal-TiO_2_ NMPs via interfacial self-assembly followed by in situ polymer deposition triggered by oil evaporation. (B) Contact angle measurement performed on Au and Au-TiO_2_ films. (C, D) Optical images showing the evolution of emulsion droplets’ color with an increasing Au-TiO_2_ ratio and the corresponding Au-TiO_2_ NMPs.

In addition to being able to assemble at the water–oil interface, the nanoparticles must also be able to stabilize oil-in-water Pickering emulsions since this is crucial for completing the in situ polymer deposition process in Step 2. This required the nanoparticles assembled at the water–oil interface to be sufficiently hydrophilic to stabilize the oil droplets from coalescing into a bulk liquid phase due to interfacial tension and capillary forces.^30,31^ Typically, colloidal metal nanoparticles do not have the appropriate surface chemistry to stabilize Pickering emulsions. As a result, Pickering emulsions formed with just Au nanoparticles at the water–oil interface coalesced immediately into an interfacial 2-dimensional array, as shown in Figure S4. In contrast, oxide nanoparticles are often sufficiently hydrophilic for stabilizing oil-in-water Pickering emulsions. Therefore, coadsorbing TiO_2_ along with Au nanoparticles created mixed nanoparticle layers, which are more hydrophilic than the pure Au nanoparticle layer. This was evident through contact angle measurements performed on flat films composed of a mixed monolayer of Au and TiO_2_ nanoparticles (Figure 1B), where the contact angle of the nanoparticle layer decreased from 68° to 15° as the wt % of Au was decreased from 100 to 1.5%. Here, it was found that stable Pickering emulsions could be obtained for samples containing around 3.5 × 10^11^ nanoparticles/mL TiO_2_ nanoparticles mixed with 20 nm Au nanoparticles up to around 8 × 10^11^ nanoparticles/mL. Increasing the concentration of Au nanoparticles above 8 × 10^11^ particles/mL destabilized the emulsions, giving a phase-separated sample in which the Au and TiO_2_ nanoparticles were packed as a two-dimensional film at the planar water–oil interface (Figure S5). Before reaching this critical point, increasing the Au-TiO_2_ ratio led to Pickering emulsions evolving from colorless to pale pink, which resembled the color of the Au colloid, and finally to dark purple, which arose from increased plasmonic coupling between the Au nanoparticles at the water–oil interface (Figure 1C).

To convert the Pickering emulsions into solid NMPs in Step 2, we took advantage of the unique biphasic properties of the oil-in-water emulsions by predissolving polystyrene in the oil phase of the Pickering emulsions. Evaporation of the dichloromethane led to gradual deposition of the dissolved polystyrene, which froze the structure of the interfacial nanoparticle assembly in situ and led to the formation of NMPs (Figure 1D). Since polystyrene is not soluble in water, deposition of polystyrene occurred only at the oil side of the water–oil interface. This is important, since this meant that the surface of the nanoparticle layer in the product NMPs was mostly exposed, with only a small portion of each of the nanoparticles physically anchored into the polystyrene, which was confirmed using scanning electron microscopy (SEM) (Figure 1A, inset, and Figure S6).

In general, the NMP powder resembled the color of the parent Pickering emulsions (Figure 1C,D), which suggested that the packing of the nanoparticle layer at the water/oil interface was largely preserved during the polymer deposition process. Closer observation of a single NMP using SEM showed that its surfaces were typically highly wrinkled (Figure 2A). This arose from the emulsion droplet shrinking during the formation of the NMPs (Discussion S1), during which the elastic nanoparticle layer wrinkled and distorted. This was confirmed with TEM studies of the cross-section of a typical NMP that showed a distorted layer of nanoparticles on the surface of the polymer core (Figure 2A, inset). In some cases, particle shedding was also observed when the reduction in the interface area became too large to be accommodated by distortion of the nanoparticle layer, as verified using ICP-MS (Table S1 and Discussion S2).

**Figure 2:**
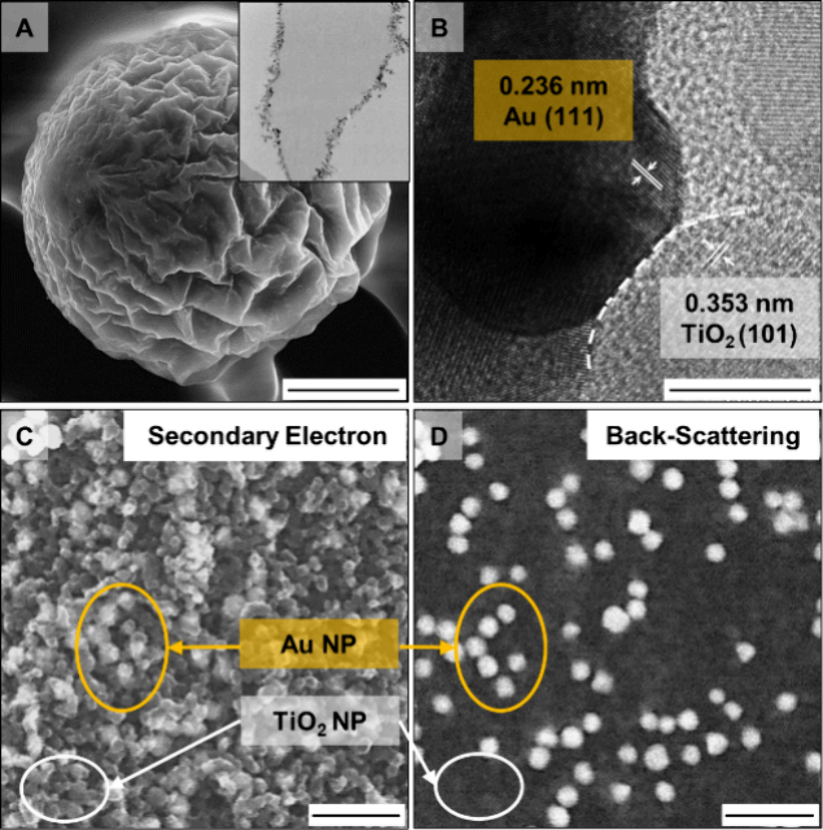
(A) SEM image of a single Au-TiO_2_ NMP showing wrinkles on its surface. The inset is the TEM image of the cross-section of a Au-TiO_2_ NMP showing a distorted thin layer of mixed Au and TiO_2_ nanoparticles on the NMP surface. (B) HRTEM showing close contact of Au and TiO_2_ nanoparticles on the NMP surface. (C, D) High-magnification SEM images of the surface of a single Au-TiO_2_ NMP. Panels (C) and (D) correspond to the same area imaged using secondary electron and backscattering detectors, respectively. Scale bars in panels (A)–(D) correspond to 50 μm, 10 nm, 200 nm, and 200 nm, respectively.

Importantly, the shrinking-to-buckling-to-particle shedding process described above ensured that the NMP formation process automatically corrected for changes in the precursor concentrations to always create tightly packed surface nanoparticle layers. This could be clearly observed in high-resolution transmission microscopy (HRTEM), which showed that the Au and TiO_2_ nanoparticles on the surface of NMPs were jammed tightly together and joined at their crystal boundaries (Figure 2B). The effective Au-TiO_2_ contact can also be confirmed using X-ray photoelectron spectroscopy (XPS), which showed an upshift of binding energy for the Ti 2p_(3/2)_ peak (Figure S15A) compared to that without Au, indicating electron transfer from TiO_2_ to Au in the Au-TiO_2_ NMPs. By comparison of SEM images produced from backscattered and secondary electrons (BSE) in Figure 2C,D, it can also be seen that the Au nanoparticles were scattered throughout the surface layer as individual nanoparticles, rather than existing as domains. Such a surface configuration can also be found for NMPs formed with TiO_2_ and other types of metal nanoparticles (such as Pt), as shown in Figures S7 and S8. This observation was consistent with our previous work on 2D interfacial assemblies formed from binary mixtures of nanoparticles, which showed that nanoparticles with similar hydrophobicity tended to mix randomly within the interfacial layers.^32^ This effect becomes less obvious at very high loading of Au (around 70 wt %), where small clusters composed of tens of Au nanoparticles could be observed. Importantly, the surface density of the noble metal nanoparticles in NMPs can be controlled by altering the Au-to-TiO_2_ ratio in the stock solution used for creating the parent emulsions. For example, as shown in Figure S9, the number density of 20 nm Au nanoparticles on the NMP surface increased linearly with the increase in the volume of Au colloid used for emulsion synthesis. This ability to fine-tune metal loading independent to other experimental parameters is not only useful for the development of new mechanistic understandings in photocatalytic hydrogen evolution shown below but also potentially useful for a range of other catalytic applications.^12,33^

Since the synthesis of NMPs did not involve any material-specific chemical processes, the self-assembly approach could be readily used to create metal–semiconductor nanocomposites with different types of nanoparticles as building blocks (Figure 3A). As a demonstration of the versatility of the self-assembly approach, Figure 3B–E and Figures S10 and S11 show a variety of NMPs formed with metal nanoparticles including Au and Pt nanospheres of different diameters, Ag polymorphic nanocrystals, Ag nanocubes, combined with oxide nanoparticles including SiO_2_ nanospheres, and P25 TiO_2_.

**Figure 3:**
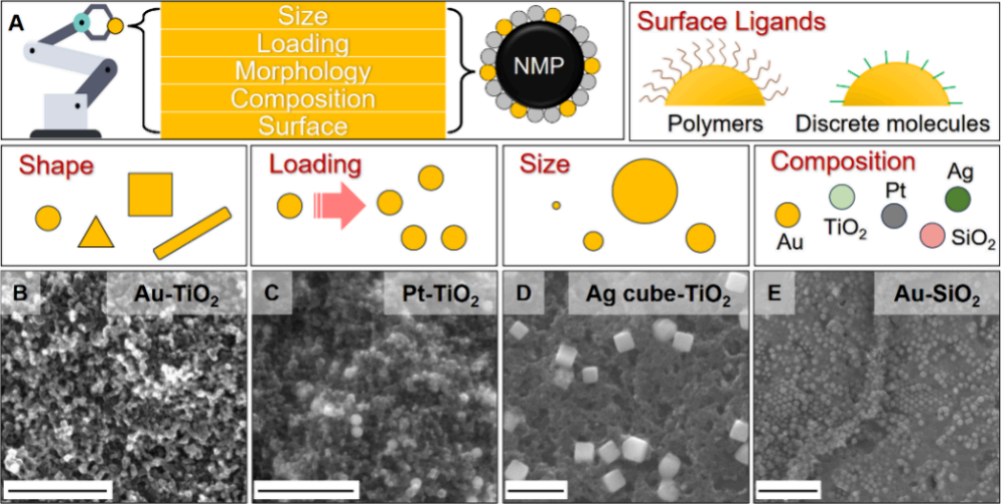
(A) Schematic highlighting the key parameters that are customizable in NMPs fabricated via the interfacial self-assembly method. (B–E) SEM images of the surface of metal–semiconductor NMPs fabricated using (B) 20 nm Au spheres and TiO_2_ nanoparticles, (C) 50 nm Pt spheres and TiO_2_ nanoparticles, (D) 180 nm Ag cubes and TiO_2_ nanoparticles, and (E) 50 nm Au spheres and SiO_2_ nanoparticles. Scale bars in panels (B)–(E) correspond to 500 nm.

Since the nanoparticles were assembled without modifying their surface with strongly adsorbed modifiers, this meant that the surface chemistry of the NMPs could be fine-tuned to study its effect on photocatalytic efficiency. In addition, the loading of the nanoparticles could also be easily adjusted by tuning the concentration of the nanoparticle building blocks used for self-assembly. This created favorable conditions for us to systematically and independently investigate the impact of various physical parameters on photocatalytic activity, which had been challenging to achieve using conventional synthetic methods. This is demonstrated below using NMPs (shown in Figure S12) formed with citrate capped quasi-spherical Pt nanoparticles of different diameters and P25 TiO_2_ nanoparticles as the model materials since Pt nanoparticles are considered to be better cocatalysts compared to Au and are one of the most widely studied cocatalysts for hydrogen evolution. As shown in Figure 3C and Figure S8, the configuration of the surface nanoparticle layer on Pt-TiO_2_ NMPs was identical to that on the surface of Au-TiO_2_ NMPs, where surface nanoparticles were packed closely against each other, with Pt nanoparticles distributed uniformly among TiO_2_ nanoparticles.

The hydrogen evolution activity of the NMPs was examined in a glycerol solution under UV excitation. Under such illumination conditions, the localized surface plasmon resonance (LSPR) of Au nanoparticles was not excited since the excitation wavelength was far from the LSPR resonance region. This enables us to investigate the cocatalytic contribution of Au nanoparticles without interference from their LSPR contributions. Glycerol was chosen as the sacrificial agent since it is an oversupplied byproduct of biodiesel manufacturing.^34,35^ As expected, the metal-TiO_2_ NMPs were found to be effective photocatalysts for hydrogen evolution due to the noble metal nanoparticles acting as a highly efficient cocatalyst, while NMPs containing just TiO_2_ were not (Figure S13).

### Probing the Effect of Surface Ligands

We start by discussing the impact of surface chemistry on photocatalytic hydrogen evolution. As discussed above, in the current approach, we avoided the use of modifiers, which allowed us to construct Pickering emulsions in which the Au/Pt nanoparticles retained weakly adsorbed citrate/chloride ligands on their surfaces. This could be characterized via surface-enhanced Raman spectroscopy (SERS) by taking advantage of the plasmonic properties of Au. Figure 4A shows the SERS spectrum obtained from the Au-TiO_2_ NMPs. It can be seen that the SERS spectrum of the Au-TiO_2_ NMPs was dominated by the vibrational bands from chloride (236 cm^–1^) and citrate ions (multiple peaks in between 1100 and 1600 cm^–1^) that were initially present on the surface of the colloidal metal nanoparticles as stabilizing agents.^36–38^ A peak at 993 cm^–1^ corresponding to polystyrene was also present in the SERS signal, since the polystyrene was physically attached to parts of the Au surface and was therefore also within the localized electromagnetic field.^39^ The SERS studies did not show vibrational bands belonging to TBA^+^NO_3_^–^ promoter molecules, which was consistent with our model that the TBA^+^NO_3_^–^ promoter molecules induced self-assembly via electrostatic interactions and did not adsorb to the surface of the nanoparticles.

**Figure 4:**
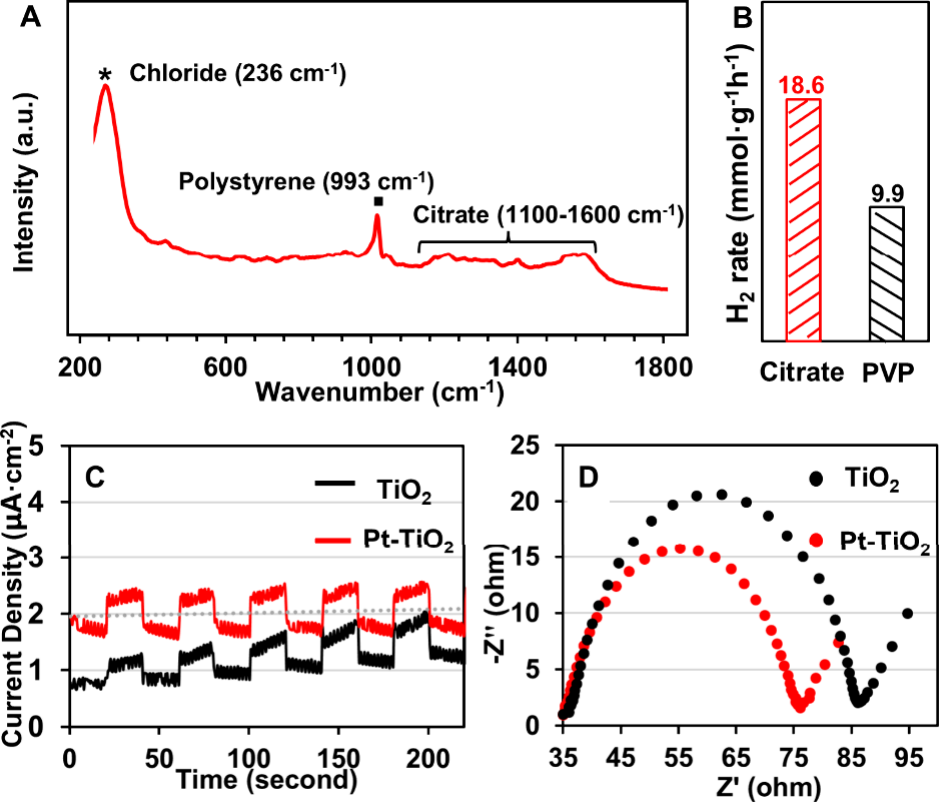
(A) SERS spectrum of Au-TiO_2_ NMPs showing signals of polystyrene citrate ions and chloride ions. (B) Histogram comparing the hydrogen evolution activity of pristine Pt-TiO_2_ NMPs and PVP-capped Pt-TiO_2_ NMPs. (C) Transient photocurrent response of Pt-TiO_2_ and TiO_2_ NMPs under irradiation by UV light showing that Pt-TiO_2_ NMPs had stronger photocurrent response. (D) Electrochemical impedance spectra of Pt-TiO_2_ and TiO_2_ NMPs showing that photoelectrons are transported more efficiently within Pt-TiO_2_ NMPs.

To study the impact of surface ligands on the efficiency of photocatalytic hydrogen evolution, different types of ligand molecules were intentionally introduced onto the surface of the nanoparticles in the NMPs. As an example, we compared the activity of pristine Pt-TiO_2_ NMPs, which are capped with citrate and chloride ligands, against the same NMPs that were intentionally treated with polyvinylpyrrolidone (PVP) ligands, since PVP is commonly used in the synthesis of noble metal catalysts.^22,23^ To study the interaction of the PVP polymer ligands with the Au, Pt, and TiO_2_ nanoparticles, zeta potential analysis was performed with the parent colloids, which allowed the surface charge of the nanoparticles to be probed in situ. As shown in Figure S14, the results showed that the Au and Pt nanoparticles carried a strong negative charge from the adsorbed citrate ligands, while the TiO_2_ colloid was slightly positively charged due to the formation of TiOH_2_^+^ under the slightly acidic conditions in our experiments. The addition of PVP led to irrelevant changes to the surface charge of the TiO_2_ nanoparticles, which was consistent with the expectation that PVP does not interact strongly with the TiO_2_ surface. In contrast, a sharp decrease in the net surface-charge value for both the Au and Pt colloids was observed with the addition of PVP, which suggested the adsorption of PVP onto the surface of the noble metal nanoparticles. To further confirm that the same effect was present when the colloidal nanoparticles were assembled into NMPs, XPS analysis was conducted to investigate the surface chemistry of the NMPs before and after treatment with PVP solution. As shown in Figure S15, the treatment with PVP gave rise to a characteristic N 1s peak, which was not present in the pristine Au/Pt-TiO_2_ NMP samples, suggesting that PVP had been successfully modified onto the nanoparticles’ surface.

Figure 4B compares the photocatalytic activity of the pristine and PVP-modified Pt-TiO_2_ NMPs, which showed that the pristine NMPs outperformed their PVP-modified counterpart by ∼2×, indicating that the surface ligands played an important role in determining the photocatalytic activity. The detrimental effect of PVP on the catalytic activity of noble metal nanoparticles has also been shown previously for other catalytic systems,^10,40^ since its bulky structure and strong affinity to metal surfaces mean that it can significantly hinder interactions between the reactants and the catalytic surface as well as metal–semiconductor interactions. In contrast, electrostatically adsorbed capping ligands, such as citrate and chloride ions, are not only more easily displaced, but also tend to occupy only ∼20% of the metal surface at full coverage, due to the effect of surface-charge accumulation.^23,41^ This is beneficial for reactant–catalyst interactions and also allows for efficient electron transfer between metal and semiconductors for more efficient separation of charge carriers. As shown in Figure 4C,D and Figure S16, metal-TiO_2_ NMPs outperformed TiO_2_ NMPs in both electrochemical impedance and photocurrent measurements, suggesting that charge separation and transfer efficiency were improved with the inclusion of metal nanoparticles. It should be noted that in addition to improving charge transfer efficiency, metal nanoparticles can also improve photocatalytic activity by providing active sites, which significantly lower the activation energy for hydrogen evolution. This might explain why a much greater difference was observed for photocatalytic activity compared to the photocurrent responses between Pt-TiO_2_ NMPs and TiO_2_ NMPs.

### Probing the Effect of Metal Loading and Nanoparticle Size

As discussed above, the loading of metal nanoparticles within the NMPs can be fine-tuned independently of other physical parameters, which allowed the effect of metal nanoparticle loading on photocatalytic efficiency to be systematically studied. Similar to the case for Au-TiO_2_ NMPs, the surface density of Pt nanoparticles in Pt-TiO_2_ NMPs was also found to be dependent on the volume ratio of the Pt and TiO_2_ colloid used. For example, as shown in Figure 5A, the surface density of 50 nm Pt nanoparticles on Pt-TiO_2_ NMPs prepared by adding 1, 3, 5, 7, and 9 mL of stock Pt colloid to the TiO_2_ solution correspond to 2.5, 6.4, 11.5, 10.6, and 26.7 number of particles per μm^2^ surface area (equivalent to 2.4, 7.2, 12.0, 16.8, and 21.6 wt %). As shown in Figure 5B, a volcano-type relationship was recorded for Pt-TiO_2_ NMPs synthesized with 50 nm Pt nanoparticles, recording highest activity when 5 mL of 50 nm Pt colloid was added (7.2 wt % Pt loading). A further increase of the loading of Pt nanoparticles led to a decrease of hydrogen evolution activity.

**Figure 5:**
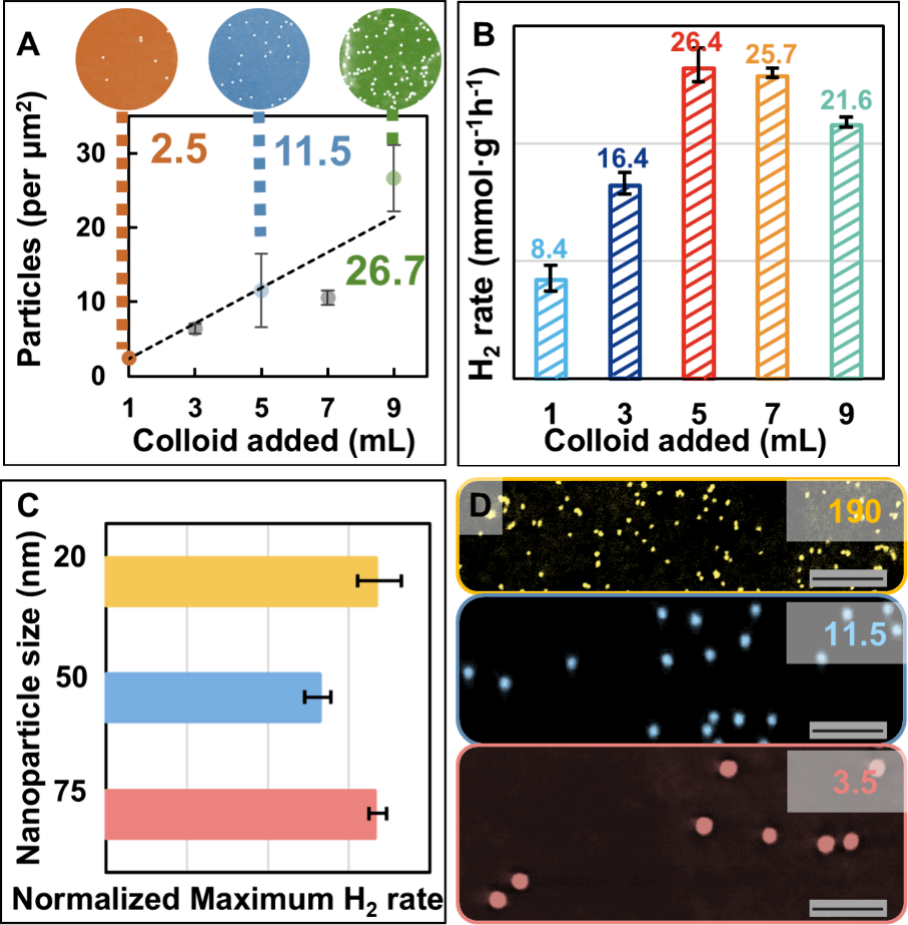
(A) Plot showing the increase of Pt density with an increasing volume of 50 nm Pt colloid added. The insets show corresponding SEM images of the Pt-TiO_2_ NMP surface. (B) Histogram showing the hydrogen evolution activity for Pt-TiO_2_ NMPs fabricated using different volumes of 50 nm Pt nanoparticles. (C) Histogram showing that the normalized hydrogen evolution activities of the best-performing Pt-TiO_2_ NMPs for each nanoparticle size were quite similar. (D) SEM image of the samples shown in panel (C). The scale bars in panel (D) correspond to 300 nm. The errors in panels (A)–(C) were calculated from three independent experiments.

In addition to metal loading, the size of the Pt nanoparticles in the NMPs can also be controlled by simply changing the size of the Pt nanoparticles used. In addition to 50 nm Pt nanoparticles demonstrated above, we also used 20 and 75 nm Pt nanoparticles for the synthesis of Pt-TiO_2_ NMPs with Pt loading, which ranged from 2.4 to 21.6 wt %. As expected, we observed that the surface densities of 20 and 75 nm Pt nanoparticles on Pt-TiO_2_ NMPs were also in direct proportion to the volume of Pt colloid added (Figure S17), and that these Pt-TiO_2_ NMPs also gave a volcano-type relationship between their activity and the loading of Pt nanoparticles (Figure S18). The corresponding apparent quantum yield value can be found in Table S2. Interestingly, although Pt-TiO_2_ NMPs synthesized with 20, 50, and 75 nm Pt nanoparticles reached the maximum activities at different loading percentages, at their, respectively, optimized loadings, the activities of 20/50/75 nm Pt-TiO_2_ NMPs were quite similar (Figure 5C). This was even more interesting considering that the density of Pt nanoparticles in the optimal NMPs changed significantly for different sizes of nanoparticles (Figure 5D). Specifically, the nanoparticle density corresponding to the maximum H_2_ production for Pt_20_-TiO_2_, Pt_50_-TiO_2_, and Pt_75_-TiO_2_ NMPs was around 190, 12, and 4 nanoparticles/μm^2^, respectively.

### Quantitative Mechanistic Modeling of Photocatalytic Activity

Although it is well established that the activities of Pt-TiO_2_ photocatalysts are significantly affected by metal loading and size, the exact reason for this remains under debate and numerous possibilities have been proposed.^16^ The effects of the experimental factors above are clearly difficult to separate since changes in one typically lead to simultaneous changes in the others during the creation of photocatalysts using conventional approaches. However, in the current study, the self-assembly method does allow such control, which make the trends observed in Figure 5 particularly interesting. To rationalize the observations presented above, we propose a model in which it is assumed that the Pt nanoparticles are in contact with the surrounding TiO_2_ nanoparticles and that each Pt nanoparticle can trap photoelectrons created by adsorption of a photon by TiO_2_ within some distance of the junctions that form at the contact points between the metal and the semiconductor (Figure 5A). This distance can be larger than the length of a single TiO_2_ crystallite because of the antenna effect, in which excitation energy can transfer between aggregated TiO_2_ nanoparticles (Figure 6B).^42,43^ If the movement of electrons within the TiO_2_ layer is a 2D random walk, the probability of trapping a photogenerated electron will be highest if it is created by adsorption of a photon by a TiO_2_ nanoparticle near the Pt electron sink but will fall off with the distance (Figure 6A). This may be represented by contours corresponding to different capture probabilities, but for current purposes, this diffusion model can be simplified by defining an area within which photogenerated electrons are captured but outside of which the trapping probability is as zero. This harvested area is enclosed by a boundary that is at a distance *R*_a_ from the particle surface. Intuitively, this could be considered to reflect the fact that the photogenerated electrons have a finite lifetime and so the boundary is set by the maximum distance that the electrons could travel before recombination.

**Figure 6:**
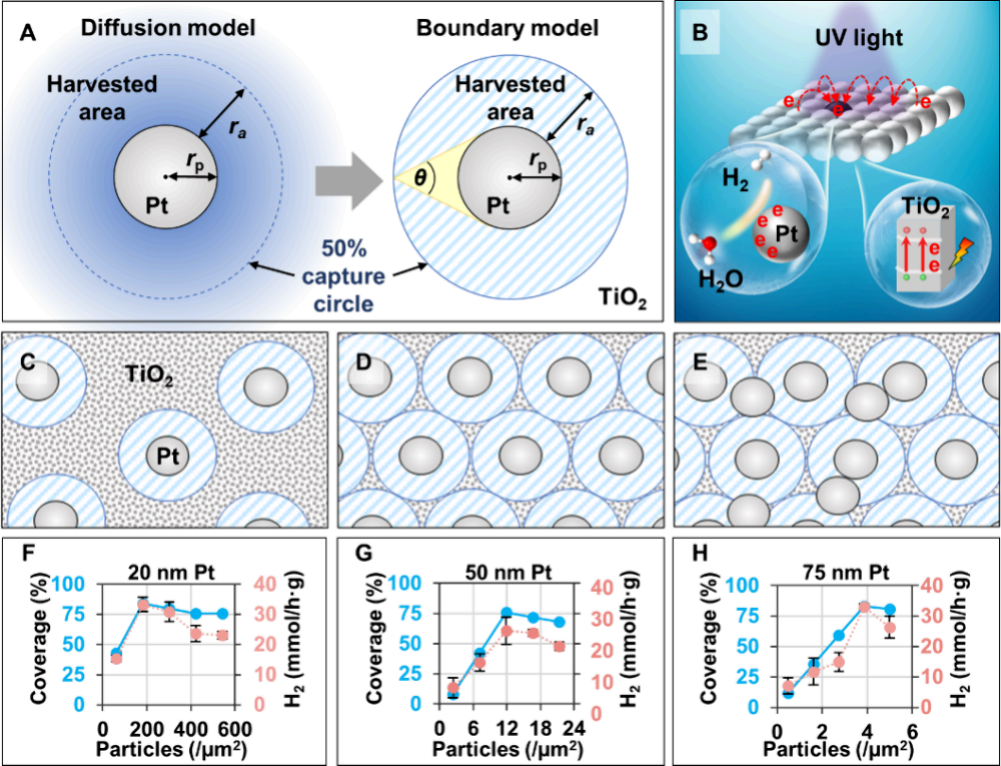
(A) Physical model illustrating the boundary conditions for effective electron capture region of a single Pt nanoparticle. (B) Schematic illustration of the hydrogen evolution process on the surface of Pt-TiO_2_ NMPs. (C–E) Schematic illustration of the Pt nanoparticle distribution on the surface of Pt-TiO_2_ NMPs with (C) *n*_p_ < *n*_max_, (D) *n*_p_ = *n*_max_, and (E) *n*_p_ > *n*_max_. (F–H) Plots comparing the modeled metal cocatalyst coverage versus experimental values for H_2_ production. The errors in panels (F)–(H) were calculated from three independent measurements.

Clearly, the boundary model implies that the rate (*r*) of the photoreaction will be proportional to the area of the surface within which photogenerated electrons will be harvested (*S*_h_). Therefore, the photoreaction rate will be expected to increase linearly with the loading of metal particles of a given diameter until the point where there are sufficient particles to ensure that all points on the surface are within the harvestable range. However, the efficiency does not plateau with the addition of further metal nanoparticles because the additional nanoparticles reduce the amount of TiO_2_ surface available for photogeneration of electrons, giving a decrease in *S*_h_ and therefore *r*, as is observed experimentally (Figure 5B). Previous models have also included the possibility that overlap of the harvested areas reduces the capture efficiency, but we have found that the data can be fitted without including this effect.^44,45^

The boundary model can be used to quantitatively describe and rationalize the relative photoreaction efficiencies observed for NMPs containing metal nanoparticles of different sizes and loadings. The starting point is to use the experimental data shown in Figure S18 to find the best estimate for the particle density that gives the maximum rate of H_2_ generation for each particle size (*n*_max_). This is assumed to be the point where the harvested area is at its maximum. Furthermore, if each nanoparticle can harvest photogenerated electrons from a circular area whose radius is given by the sum of the particle radius (*R*_p_) and an antenna length (*R*_a_) and the number of particles (*n*_*p*_) per unit area is known, then the radius of the harvested area (*R*_p_ + *R*_a_) required for the maximum packing (0.90 of available area for hexagonally close packed circles) can also be calculated. This gives the *R*_a_ for each *R*_p_. Detailed calculation of *R*_a_ can be found in Discussion S3. The area of the surface from which photogenerated electrons are harvested (*S*_h_) can be calculated by using eq 1.

$$S_{h}=\pi n_{p}((R_{p}+R_{a}{)}^{2}-R_{p}^{2})$$ 1

*S*_h_ is the total area enclosed by the boundary at distance *R*_a_, from the perimeter of the particles, minus a correction for the reduction in photocatalyst area due to Pt occupying some of the surface instead of TiO_2_, which is a straightforward geometrical factor equal to *n*_p_π*R*_p_^2^. However, this expression for *S*_h_ is only correct if *n*_p_ ≤ *n*_max_, since at *n*_p_ > *n*_max_, additional nanoparticles do not give increased harvesting but they do continue to reduce the amount of the TiO_2_ surface available for photogeneration of electrons (see schematics in Figure 6C–E). This means that the *n*_p_π*R*_p_^2^ correction will continue to increase above *n*_max_. So, at *n*_p_ > *n*_max_, *S*_h_ can be calculated following eq 2.

$$S_{h}=\pi n_{\max}(R_{p}+R_{a}{)}^{2}-n_{p}\pi R_{p}^{2}$$ 2

Therefore, in general, once the values of *R*_p_ and *R*_a_ are known, *S*_h_ can be calculated for any given surface density of particles of that size following the equations above.

Figure 6F–H includes plots of *S*_h_ vs *n*_p_ for the three Pt-TiO_2_ series along with the experimental data for *r* (the rate of H_2_ generation) vs *n*_*p*_, which is also shown in Figure 5B. The agreement of the predicted and experimental data is striking, especially given that the model does not include any adjustable fitting parameters. This gives us confidence that the physical assumptions underpinning the model are appropriate and do accord with the underlying physical processes taking place in real systems. The values for *n*_p_ and *R*_a_ for each *R*_p_ are given in Table 1. These values show that in the Pt_50_-TiO_2_ NMP series for example, since *n*_max_ is around 12 nanoparticles/μm^2^, the average center-to-center distance between nanoparticles is around 300 nm, demonstrating that the harvesting can occur over lengths considerably greater than the particle diameter. More importantly, this also suggests that the size effect, which is known to influence the electronic properties of Pt nanoparticles and therefore, the ability of Pt to accept electrons from TiO_2_, might not be the decisive factor in determining the overall hydrogen evolution activity for the current Pt-TiO_2_ system.^16^

**Table 1:** Table Showing How the Antenna Length (*R*_a_), Particle Density Giving the Maximum Rate of H_2_ Generation (*n*_max_), and Particle Radius (*R*_p_ *)*, Varied for the Three Different Pt-TiO_2_ NMPs Investigated

parameter	*R*_a_ (nm)	*n*_max_ (particles μm^–2^)	*R*_p_ (nm)
Pt20-TiO_2_	29	190	10
Pt50-TiO_2_	130	12	25
Pt75-TiO_2_	230	4	37.5

The model revealed that *R*_a_ values change dramatically for different *R*_p_ values and that larger nanoparticles harvest electrons from much larger regions. This finding, although somewhat counterintuitive, reflects the data in the SEM images of the NMPs that gave the maximum H_2_ production, where, for example, the Pt-TiO_2_ NMP formed with 75 nm Pt nanoparticles had peak efficiency at only ca. 4 particles/μm^2^. The changes in *R*_a_ with *R*_p_ can be rationalized by considering that the probability of electron capture will depend on the capture angle, θ, as shown in Figure 6A. Detailed calculations of θ can be found in Discussion S4. To maintain constant θ with varying *R*_p_, the ratio of the sum of *R*_a_ + *R*_p_ to *R*_p_ should stay the same. This leads to the prediction that ratios of the *R*_a_ values for the Pt-TiO_2_ NMPs formed with 20, 50, and 75 nm Pt nanoparticles would be 0.4:1.0:1.5, which matches well to the ratios of 0.3:1.0:1.7 calculated from the experimental *R*_a_ values shown in Table 1. This suggests that the physical assumptions underpinning the model are appropriate and capture the main features of the underlying mechanism.

### Rational Design of Metal–Semiconductor Photocatalysts

The discussion above emphasizes the advantage of having control over the materials for developing models that allow a deeper understanding of the photocatalytic reaction. Using this approach also has the practical outcome of enabling the construction of effective photocatalytic materials. In the current study, the hydrogen evolution rate of the NMPs formed using common citrate-stabilized metal colloids and P25 nanoparticles was superior to that of many carefully designed state-of-the-art composite materials (Table S3). As shown in Figure S20, the activity of Pt-TiO_2_ NMPs also outperformed that of TiO_2_ NMPs, which were photodeposited with the same amount of Pt, demonstrating the potential of the current self-assembly method for synthesizing high-performance photocatalysts. Moreover, since the metal-TiO_2_ nanoparticles are partially embedded within the polystyrene matrix in NMPs as a near-monolayer, this maximizes the utilization efficiency of nanoparticles while preventing them from shedding and aggregating during use. This allowed the NMPs to sustain high activity for at least five repeated cycles over a total period of 300 min (Figure S19).

## Conclusions

In conclusion, we have presented a convenient and general emulsion-templated self-assembly strategy to fabricate powder-type metal-TiO_2_-polymer composite materials termed NMPs using nanoparticles of different morphologies, compositions, and surface chemistry. Unlike conventional strategies to fabricate metal-TiO_2_ photocatalysts, where the aforementioned critical parameters are intertwined, our method allowed independent control over each parameter, which paved the way for systematic investigations of how different material properties contributed to the overall hydrogen evolution activity of the photocatalyst. Most importantly, our studies revealed that the optimal particle loading varied by several orders of magnitude for metal nanoparticles of different diameters and that the overall activity of Pt-TiO_2_ NMPs is determined by the size of the area from which photogenerated electrons are harvested. This unexpected phenomenon can be explained by considering the capture angle of photoinduced electrons by the nanoparticles, which is dependent on the size of the metal nanoparticle and distance the electrons travel before reaching the metal. This new understanding combined with the versatility of the self-assembly method meant that the product metal-TiO_2_ photocatalysts can be easily and rationally optimized to achieve high activity. This was demonstrated by systematically controlling the loading of the metal nanoparticles, which produced Au/Pt-TiO_2_ NMPs that outperformed carefully designed state-of-the-art composite materials in hydrogen evolution. More broadly, the self-assembly approach can be readily extended to a wide range of metal and semiconductor materials, which gives it potential to be applied across a broad range of applications including sensing and catalysis.

## Methods

### Materials

Silver nitrate (99.9999%), gold­(III) chloride trihydrate (99.9999%), chloroplatinic acid hexahydrate, trisodium citrate, tetrabutylammonium nitrate (TBA NO_3_), dichloromethane (DCM), sodium borohydride, l-ascorbic acid, and screw-cap polypropylene centrifuge tubes were purchased from Aldrich Ltd. Polystyrene (M.W. ≈ 100,000) was purchased from BDH chemicals Ltd. Silica nanospheres (50 nm) (10 mg/mL) were purchased from nanoComposix. P25 TiO_2_ colloid (0.4 g/mL) was purchased from Evonik. All chemicals were used without further purification. DDI water used throughout all experiments had low total organic content (<3.0 ppb) 18.2 MΩcm.

### Au Colloid Synthesis

Citrate-reduced Au colloid was synthesized using a previously reported seed-mediated method with slight modification.^46^ Briefly, 1 mL of HAuCl_4_ (25 mM) was added into 150 mL of sodium citrate water solution (2.2 mM) at 100 °C. The mixture was kept boiling for 30 min to complete the synthesis of gold seed nanoparticles with a diameter around 10 nm. After that, the mixture was cooled to 90 °C. Then, 1 mL of sodium citrate (60 mM) and 1 mL of HAuCl_4_ solution (25 mM) were sequentially injected (time delay ∼2 min). The mixture was allowed to react for another 30 min at 90 °C. By repeating this process 3 times, 6 times, and 9 times (sequential addition of 1 mL of sodium citrate 60 mM and 1 mL of HAuCl_4_ 25 mM), Au nanoparticles with a size around 20, 30, and 40 nm were synthesized. The size distribution of as-prepared Au nanoparticles can be found in Figure S1.

### Pt Colloid Synthesis

Citrate-reduced Pt colloid was synthesized using previously reported seed-mediated method with slight modification.^47^ Briefly, the Pt seed was prepared by adding 3 mL of HPtCl_6_·H_2_O solution (0.2%) and 0.92 mL of sodium citrate (1%) solution in sequence (with 1 min delay) to 39 mL of boiling DDI water. This was followed by adding 0.46 mL of freshly prepared sodium borohydride solution (with 0.5 min delay). The mixture was allowed to react for 10 min for the formation of Pt seed. The seed solution was cooled down to room temperature.

For the synthesis of 20 (50) (75) nm Pt nanoparticles, 1 mL of as-prepared Pt seed (20 nm Pt nanoparticles) (50 nm Pt nanoparticles), 0.045 mL of HPtCl_6_·H_2_O solution (0.4 M), and 0.5 mL of l-ascorbic acid solution (1.25 wt %) containing sodium citrate (1 wt %) were added in sequence to 30 mL of DDI water at room temperature. The solution was heated to boil and left to react for 30 min to complete the synthesis. The SEM images and size distribution of as-prepared Pt nanoparticles can be found in Figure S2.

### Ag Colloid Synthesis

Citrate-reduced Ag colloid was synthesized using the Lee and Meisel method.^48^ In a typical experiment, 45 mg of AgNO_3_ was dissolved in 250 mL of DDI water, followed by heating and stirring under reflux until a boiling temperature was reached. When boiling, 5 mL of 1 wt % aqueous trisodium citrate was added in continuously over 30 s with a syringe. The mixture was then allowed to react for another 90 min, where it would gradually change from being transparent and colorless to cloudy and grayish green before being cooled down to room temperature.

### Fabrication of Metal-TiO_2_ NMPs

The concentration of polystyrene/DCM solution was 0.1 g/mL. The concentration of TBA NO_3_ solution was 1 mM. TiO_2_ colloid was diluted by a factor of 80 to reach a final concentration of 5 mg/mL. Before fabrication, the mixture of TiO_2_ colloid and Au (Pt) colloid was diluted or concentrated into 20 mL. This was then shaken with DCM and TBA NO_3_ solutions, to create emulsion droplets, which were left undisturbed at room temperature for at least 3 days to obtain solid metal-TiO_2_ photocatalysts. The exact volume of each solution differed depending on the experiment and is described in detail in Table S4.

### Fabrication of Au-TiO_2_-Polystyrene Films

The Au-TiO_2_ films were synthesized following the method previously reported by our group.^39,49^ In brief, to synthesize Au nanoparticle films, 1 mL of Au colloid synthesized using the Frens method was diluted to 5 mL by adding 4 mL of DDI water. The diluted colloid was shaken with 3 mL of polystyrene/dichloromethane (0.05 g mL^–1^) solution and 0.12 mL of tetrabutylammnonium nitrate (10^–3^ M) for 30 s. The mixture was poured immediately into a polypropylene Petri dish. A liquid metal film was formed after the emulsion droplets coalesced. The film was fixed on a polystyrene film after dichloromethane evaporated completely, giving rise to a freestanding Au film composed of a monolayer of Au nanoparticles and a polystyrene support. Au-TiO_2_ films were synthesized by replacing 1 mL of Au colloid with 1 mL of TiO_2_ colloid and 0.15 (0.3) mL of Au colloid.

### Photocatalytic Hydrogen Evolution from Glycerol Solution

In a typical experiment, 120 mg of photocatalyst was suspended in 30 mL of a predetermined concentration of glycerol (typically 1 M) inside a small glass reaction vessel. The glass reactor had an operating volume of 30 mL and a gas headspace of 70 mL with two sample ports that were sealed with rubber stoppers. A magnetic stir bar was used to continuously stir the reaction suspension. Prior to any irradiation, the reaction suspension was purged with N_2_ for 20 min in the dark at a flow rate of 20 mL min^–1^ to remove O_2_. Irradiation of the reactor was provided by a bespoke cylindrical UV-LED array that provided 360° irradiation of the suspension. The LED strips used in the array (Lighting Will) were operated with a *V*_F_ = 12 dcV and an *I*_F_ = 0.12 A, which gave an overall power consumption of 1.44 W. The LEDs had a peak wavelength in the range 365–370 nm.

### Instrumentation

Photocurrent measurements were performed using a standard three electrode cell on an electrochemical workstation (Zahner, Zennium). The working electrode was prepared by depositing NMPs on a fluoride-tin oxide glass slide. A Pt wire was used as the counter electrode, and a saturated calomel electrode was used as the reference electrode. An aqueous solution of 0.5 M Na_2_SO_4_ was used as the electrolyte solution, and a 300 W Xe lamp equipped with a UV filter was used as the light source.

Electrochemical impedance measurements were also performed by using a standard three electrode cell on an electrochemical workstation (Zahner, Zennium). The working electrode was prepared by depositing NMPs on a fluoride-tin oxide (FTO) glass slide. A Pt wire was used as the counter electrode, and a saturated calomel electrode was used as the reference electrode. A mixture solution of 25 mM K_3_Fe­(CN)_6_, 25 mM K_4_Fe­(CN)_3_, and 0.1 M KCl was used as the electrolyte solution.

SEM analysis was performed using a Quanta FEG 250 instrument at an acceleration voltage of 20 kV under high chamber vacuum (8 × 10^–5^ mbar). SEM samples were attached on standard SEM copper tape or carbon tape as background. HRTEM was performed using a JEM-2100 at an accelerating voltage of 200 kV.

SERS spectra were collected with a PerkinElmer RamanMicro 200 Raman Microscope equipped with a 785 nm diode laser by using a total accumulation time of 20 s and 200 mW laser power.

XPS analysis was performed using a ESCALAB 250Xi system (Thermo Scientific) with a monochromated Al Kα radiation X-ray source operating at 5 × 10^–7^ mbar and 72 W. Survey scans were acquired at an analyzer pass energy of 100 eV in 1 eV steps, while high-resolution narrow scans were performed at a constant pass energy of 50 eV in 0.05 eV steps.

Zeta potential analysis was performed on a ZetaSizer Nano instrument (Malvern Instruments). Pt, Au, and TiO_2_ colloids were all diluted by a factor of 10 before being transferred to a DST1070 cell and measured at 25 °C. A viscosity of 0.8872 cP, a dielectric constant of 78.5, and a refractive index of 1.33 were used for calculations.

## Supplementary Material



## Notes

Additional experimental details, characterization results, and references; addition experimental data on materials synthesis and characterization including particle size distribution, SEM analysis, EDX analysis, XPS analysis, and zeta potential analysis; and additional data on the photocatalytic properties including catalytic efficiency, transient photocurrent response, electrochemical impedance spectra, and quantum yield (PDF)
