# Supplementary material for: Disentangling the Effect of Key Parameters in Hydrogen Evolution for Rational Design of Metal–Semiconductor Photocatalysts via Self-Assembly
Source: ACS Appl Mater Interfaces. 2025 Aug 14;17(34):49069–78. doi: 10.1021/acsami.5c14789 (PMC12400263; doi:10.1021/acsami.5c14789)
Supplement: Supplementary file 1 [file am5c14789_si_001.pdf]

**Supporting Information**

# Disentangling the effect of key parameters in hydrogen evolution for rational design of metal-semiconductor photocatalysts via self-assembly

Chunchun Li[a,c], Ziwei Ye[b*], Shan Xu[b], Nathan Skillen[c], Yingrui Zhang[c], Zehong Xu[b], Colby Chang[c], Jinlong Zhang[b], Peter K. J. Robertson[c], Steven. E. J. Bell[c*] and Yikai Xu[b*]

[a] Dr. C. Li

School of Materials Science and Engineering, East China University of Science and Technology, Shanghai, P. R. China, 200237.

[b] Dr. Z. Ye, Ms. S. Xu, Mr. Z. Xu, Prof. J. Zhang, Dr. Y. Xu

Key Laboratory for Advanced Materials and Feringa Nobel Prize Scientist Joint Research Center, Frontiers Science Center for Materiobiology and Dynamic Chemistry, School of Chemistry and Molecular Engineering, East China University of Science and Technology, 130 Meilong Road, Shanghai, P. R. China, 200237.

[c] Dr. C. Li, Dr. N. Skillen, Dr. Y. Zhang, Mr. C. Chang, Prof. P. K. J. Robertson, Prof. S. E. J. Bell

School of Chemistry and Chemical Engineering, Queen’s University Belfast, University Road, Belfast, Northern Ireland, UK, BT9 5AG.

Corresponding Author

Yikai Xu, Email address: yikaixu@ecust.edu.cn

Steven. Bell, Email address: s.bell@qub.ac.uk

Ziwei Ye, Email address: yeziwei@ecust.edu.cn

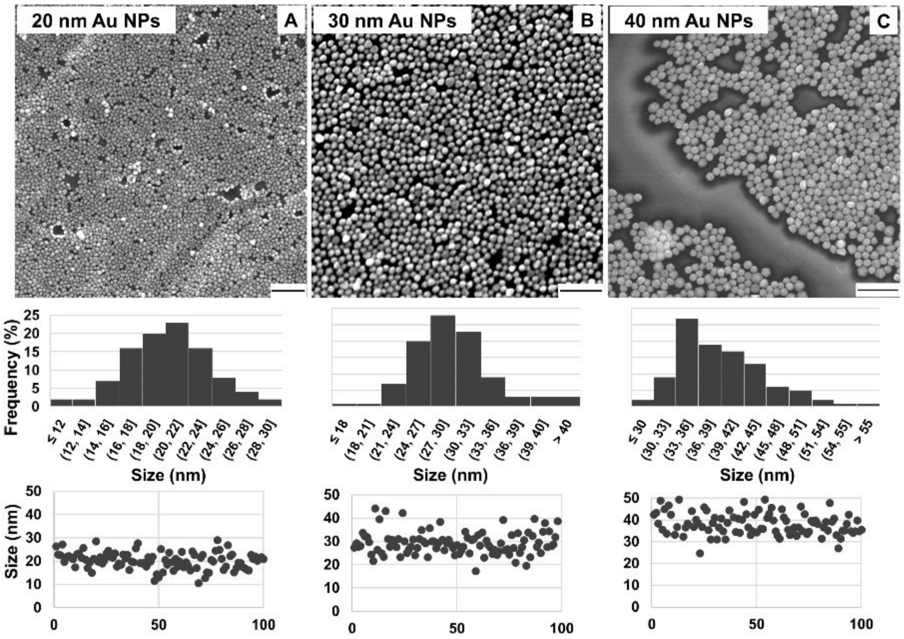


**Figure S1.** (A-C) SEM images of quasi-spherical (A) 20, (B) 30, and (C) 40 nm Au nanoparticles used in NMP formation and charts illustrating their size distribution. All scale bars correspond to 200 nm.

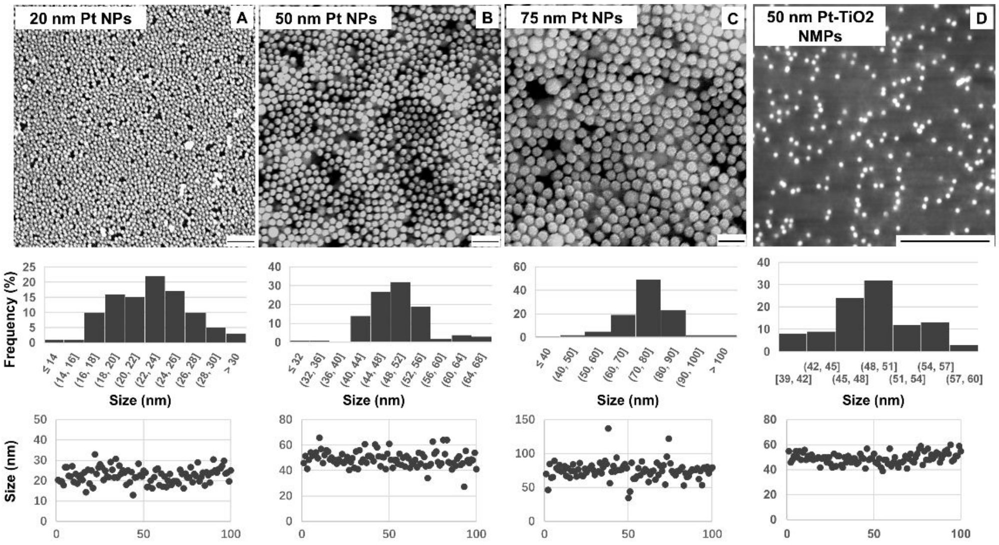


**Figure S2.** (A-C) SEM images of quasi-spherical (A) 20, (B) 50, and (C) 75 nm Pt nanoparticles used in NMP formation and charts illustrating their size distribution. (D) SEM images of Pt-$TiO_2$ NMPs synthesized with quasi-spherical 50 nm Pt nanoparticles and charts illustrating the size distribution of 50 nm Pt nanoparticles after the synthesis of Pt-$TiO_2$ NMPs. The scale bars in (A-C) correspond to 200 nm. The scale bar in D corresponds to 1 μm.

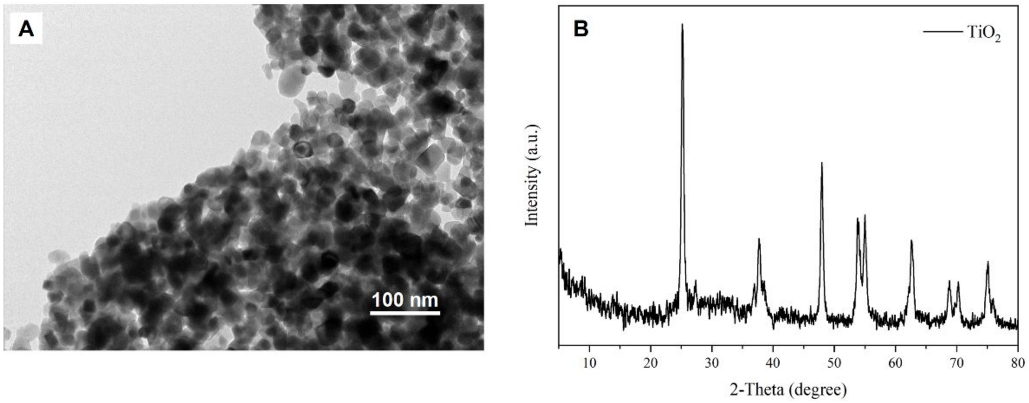


**Figure S3.** (A) HRTEM images of $TiO_2$ (type P25) nanoparticles used in NMP formation. (B) XRD spectrum of $TiO_2$ (type P25) nanoparticles used in NMP formation.

As shown in Figure S3, the shape of $TiO_2$ nanoparticles is irregular with their size varying from 10 nm to 40 nm. In addition, XRD characterization revealed that these are typical P25 nanoparticles containing a mixture of rutile and anatase phase.

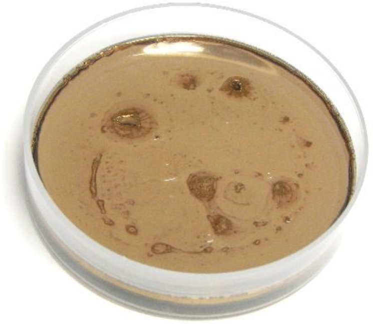

**Figure S4**. Optical image of Au arrays formed at the interface of water and dichloromethane.

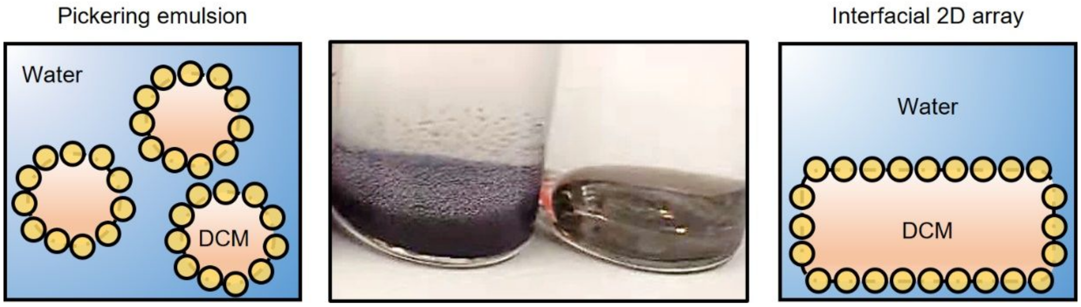


**Figure S5.** Schematic illustrations and optical images of Pickering emulsions and interfacial arrays formed with different $TiO_2$-to-Au ratio.

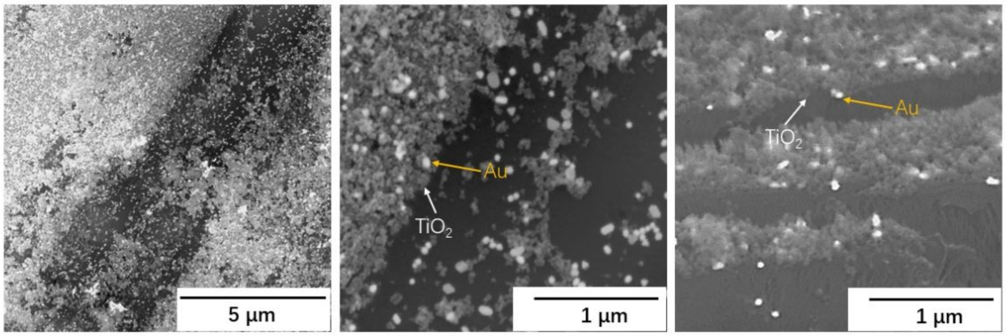


**Figure S6.** Top view (left and mid) of the surface nanoparticle layer on a typical Au-$TiO_2$ NMP. Tilted view (right) of the same area showing that the particles are present as a near-monolayer anchored on the polymer. The imaging was deliberately performed on an area with low particle density to reveal the polymer beneath.

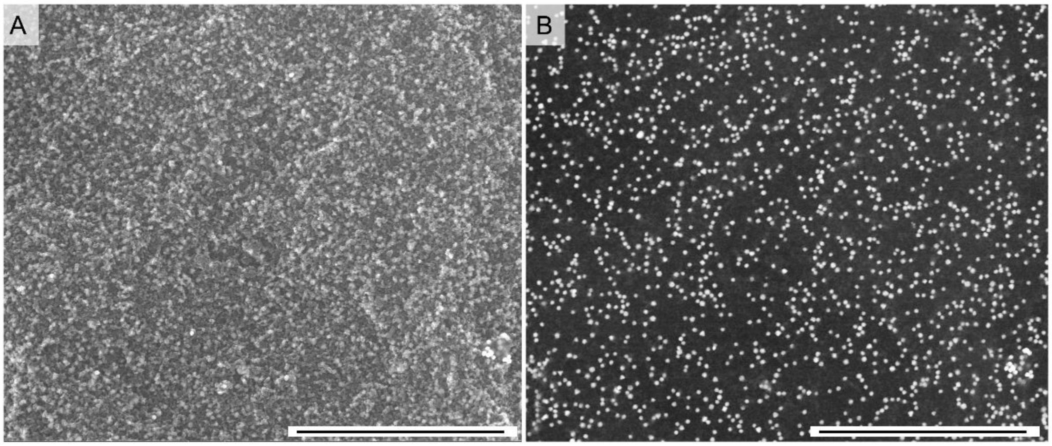


**Figure S7**. (A) Secondary electron and (B) backscattering SEM images for typical Au-$TiO_2$ NMPs formed with 40 nm Au nanoparticles. The scale bars in (A) and (B) correspond to 2 µm.

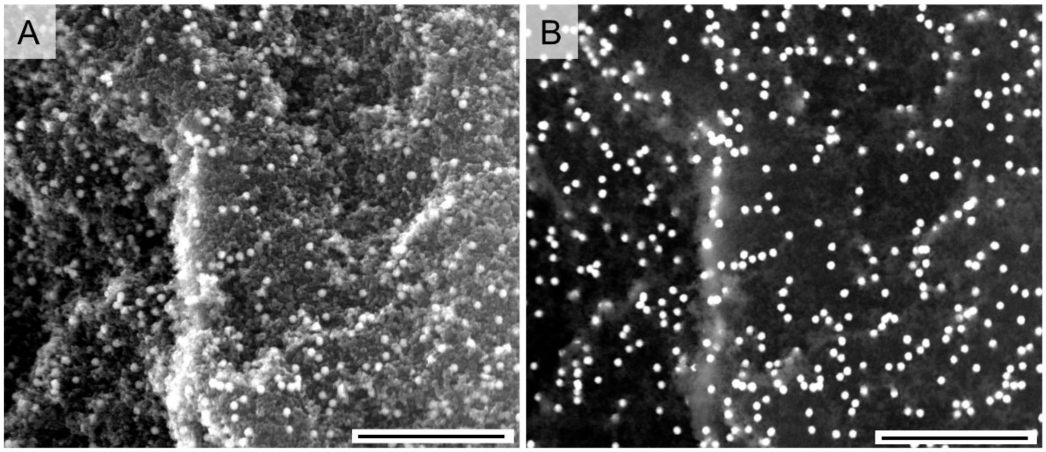


**Figure S8**. (A) Secondary electron and (B) backscattering SEM images for typical Pt-$TiO_2$ NMPs formed with 50 nm Pt nanoparticles. The scale bars in (A) and (B) correspond to 1 µm.

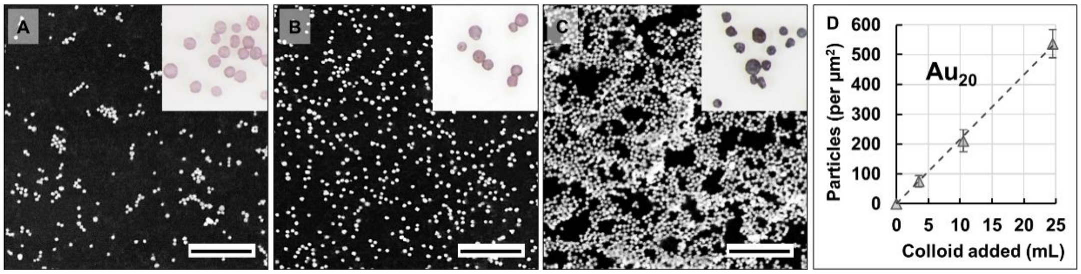


**Figure S9.** (A)-(C) SEM images of Au-$TiO_2$ NMPs showing different surface density of Au nanoparticles. (D) Plot showing the linear relationship of the surface density of Au nanoparticles with the amount of Au colloid added. The scale bars in (A)-(C) correspond to 400 nm.

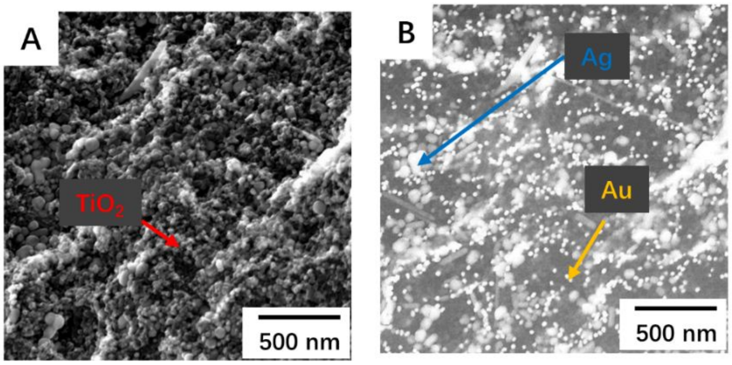


**Figure S10**. (A) SEM image of Au-Ag-$TiO_2$ NMPs taken using the secondary-electron detector. (B) SEM image of the same spot shown in A taken using the back-scattering detector.

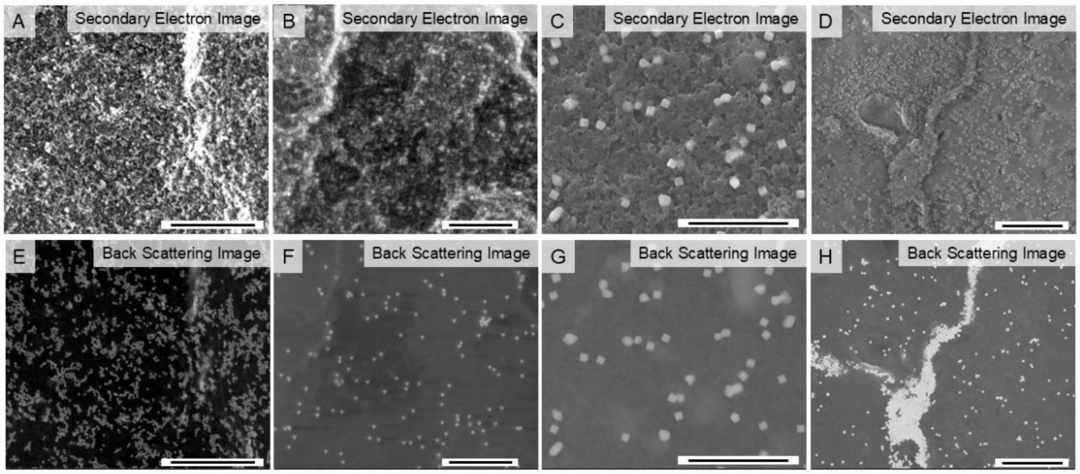


**Figure S11**. Secondary electron and backscattering SEM images showing larger area of the same sample shown in Figure 3B: (A and E) Au-$TiO_2$ NMPs; (B and F) Pt-$TiO_2$ NMPs; (C and G) Ag-$TiO_2$ NMPs; (D and H) Au-$SiO_2$ NMPs. The scale bars in (A), (B), (D), (E), (F) and (H) correspond to 1 µm. The scale bar in (C) and (G) correspond to 2 µm.

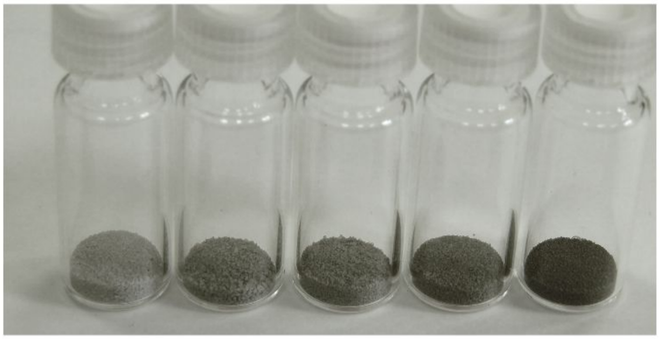

**Figure S12**. Optical image of Pt-$TiO_2$ NMPs with different Pt loadings.

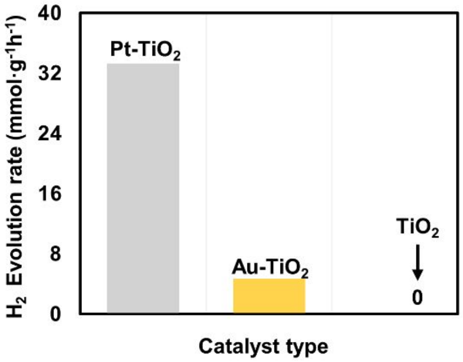


**Figure S13.** Histogram comparing the hydrogen evolution rate of the best Pt-$TiO_2$ NMPs, the best Au-$TiO_2$ NMPs, and pure $TiO_2$ NMPs.

In the current study, hydrogen evolution rate normalized by weight was used for comparing the performance of different photocatalysts. This is because at low photocatalyst concentration (0.4 mg of effective photocatalyst was used for reaction), hydrogen evolution rate would be proportional to the amount of photocatalyst used. [1]

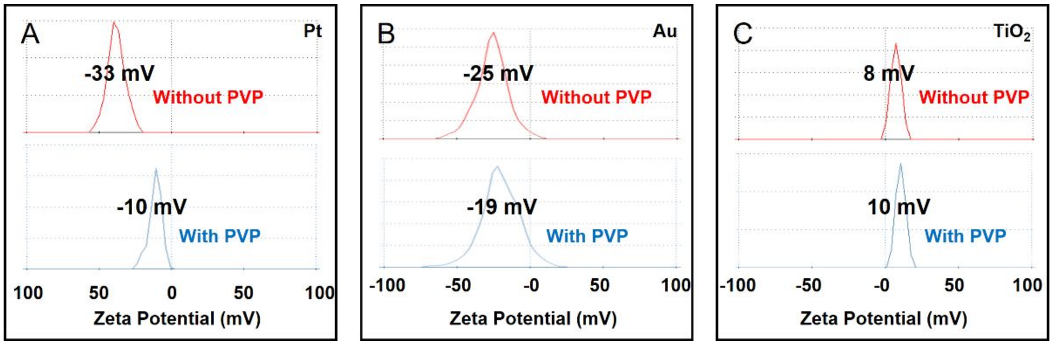


**Figure S14**. Zeta potential analysis of the (A) Pt colloid, (B) Au colloid and (C) P25 $TiO_2$ colloid used for NMP synthesis before and after being treated with PVP.

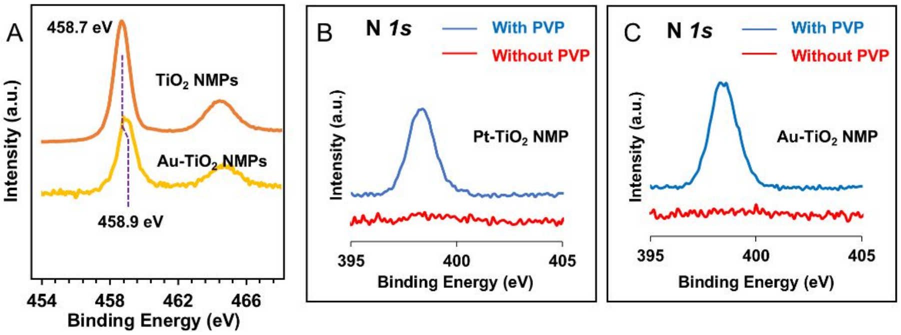


**Figure S15.** (A) XPS analysis of Au-$TiO_2$ NMPs showing the upshift of Ti 2p peaks compared to that for $TiO_2$ NMPs, indicating electron transfer from $TiO_2$ to Au in Au-$TiO_2$ NMPs. (B,C) XPS spectra of Pt/Au-$TiO_2$ NMPs before and after PVP modification in the region of N 1*s*.

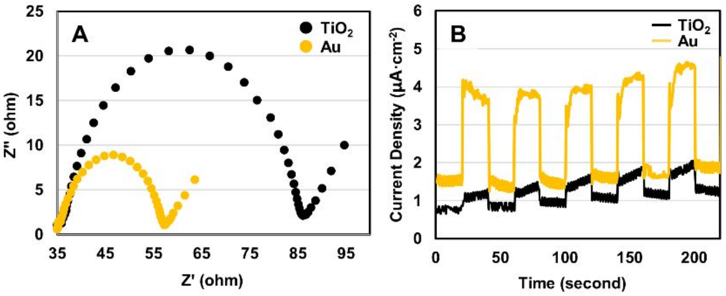


**Figure S16**. (A) Electrochemical impedance spectra of Au-$TiO_2$ and $TiO_2$ NMPs showing that photoelectrons are transported more efficiently within Au-$TiO_2$ NMPs. (B) Transient photocurrent response of Au-$TiO_2$ and $TiO_2$ NMPs under irradiation of UV light showing that Au-$TiO_2$ NMPs had stronger photocurrent response.

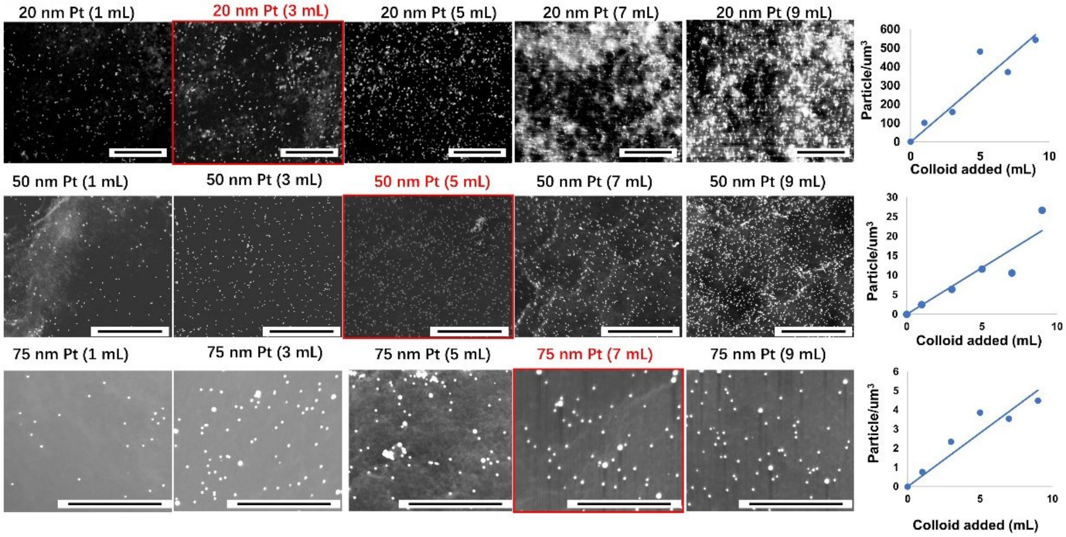


**Figure S17.** SEM images of Pt-$TiO_2$ NMPs fabricated with Pt nanoparticles of different sizes and loadings. The scale bars for 20 nm Pt correspond to 500 nm. The scale bars for 50 and 75 nm Pt correspond to 3μm.

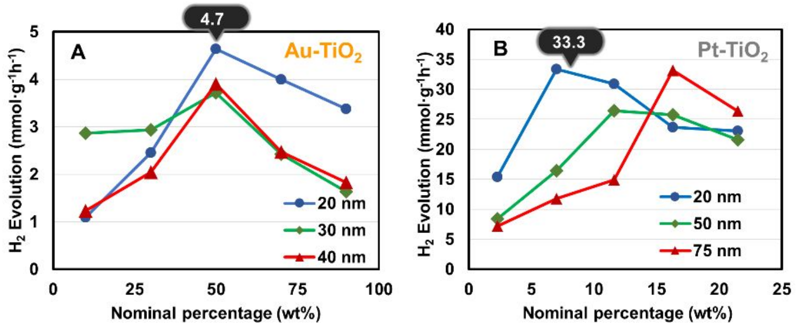


**Figure S18.** Plots showing the activity of (A) Au-$TiO_2$ and (B) Pt-$TiO_2$ NMPs at different sizes and loadings.

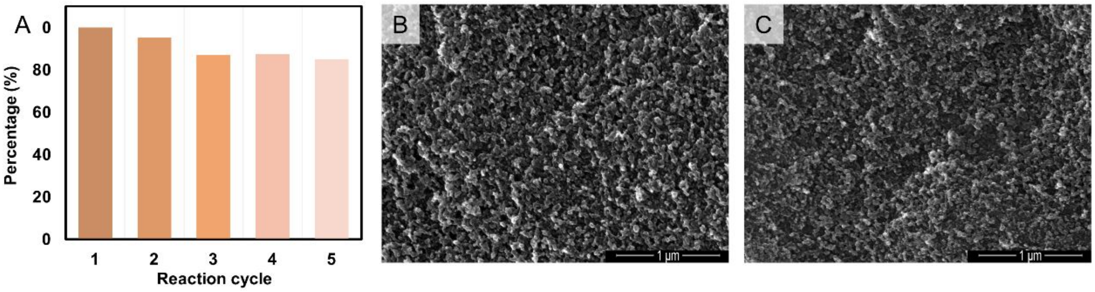


**Figure S19.** (A) Histogram showing activity of Pt-$TiO_2$ NMPs for 5 cycles of photocatalytic hydrogen evolution reaction. (B-C) SEM images of the surface nanoparticle layer on a Pt-$TiO_2$ NMP before (B) and after (C) 5 cycles of hydrogen evolution showing that nanoparticles remain tightly fixed on the polystyrene core.

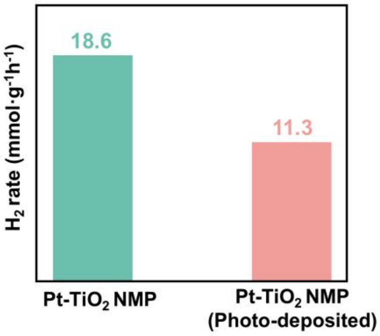


**Figure S20.** Histogram comparing the activity of Pt-$TiO_2$ NMP with $TiO_2$ NMPs photodeposited with Pt under identical experimental conditions.

| | 1st | 2nd | 3rd | 4th | 5th | 6th | Average | Theoretical |
|---|---|---|---|---|---|---|---|---|
| Pt | 0.064 wt% | 0.047 wt% | 0.071 wt% | 0.062 wt% | 0.077 wt% | 0.058 wt% | 0.063 wt% | 0.07 wt% |
| $TiO_2$ | 0.32 wt% | 0.24 wt% | 0.27 wt% | 0.32 wt% | 0.45 wt% | 0.40 wt% | 0. 33 wt% | 1 wt% |

**Table S1.** ICP analysis of the Pt-$TiO_2$ NMPs formed with 3 mL of 20 nm Pt colloid showing that the amount of $TiO_2$ on NMPs was less than that used for preparing Pickering emulsions.

| 20 nm Pt-$TiO_2$ NMPs | Pt colloid added | AQY | 50 nm Pt-$TiO_2$ NMPs | Pt colloid added | AQY | 75 nm Pt-$TiO_2$ NMPs | Pt colloid added | AQY |
|---|---|---|---|---|---|---|---|---|
| | 1 | 4.6 | | 1 | 2.5 | | 1 | 2.1 |
| | 3 | 10.0 | | 3 | 4.9 | | 3 | 3.5 |
| | 5 | 9.3 | | 5 | 7.9 | | 5 | 4.5 |
| | 7 | 7.1 | | 7 | 7.7 | | 7 | 9.9 |
| | 9 | 6.9 | | 9 | 6.5 | | 9 | 7.9 |

**Table S2**. AQY values for Pt-$TiO_2$ NMPs of different Pt size and loading percent.

In this study, UV LED was used as the light source with a very narrow spectra output in the range from 365 to 370 nm. The apparent quantum yield (AQY) of NMPs at 370 nm can be calculated using the following function.

$$\text{AQY}(\%) = \frac{Number\ of\ reacted\ electrons}{Total\ number\ of\ incident\ photons} \times 100 \qquad (1)$$

$$= \frac{2 \times The\ number\ of\ evoluted\ H_2\ molecules}{Total\ number\ of\ incident\ photons} \times 100 \qquad (2)$$

| Photocatalyst | Light source | HER (mmol/g *h) | Sacrificial agent | Reaction temperature (°C) | Ref |
|---|---|---|---|---|---|
| $TiO_2$-Pt NMPs | four 1.44W LED light (λ=365-370 nm) | 33.3 | 1 M glycerol | Room temperature | This work |
| $TiO_2$-Au NMPs | four 1.44W LED light (λ=365-370 nm) | 4.3 | 1 M glycerol | Room temperature | This work |
| $TiO_2$-Au-$MoS_{2+x}$ | four 3W LED light (λ=365 nm) | 7.8 | 25 vol.% ethanol | Not provided | 2 |
| $TiO_2$-Au-$NiS_{1+x}$ | four 3W LED light (λ=365 nm) | 9.6 | 25 vol.% ethanol | Not provided | 3 |
| $TiO_2$-Pt | four 20W Mercury Tubes | 4.1 | 5.4 M glycerol | 25 ℃ | 4 |
| $TiO_2$-Pt-Cu | 300W Xe lamp | 10 | 1.4 M glycerol | 25 ℃ | 5 |
| $TiO_2$-Pt-$SrSO_4$ | Simulated sunlight, 300 $mW/cm^2$ | 10.5 | 20 vol.% methanol | Not provided | 6 |
| $TiO_2$-Pt (single atom) | 300W Xe lamp | 13.5 | 20 vol.% methanol | Not provided | 7 |
| $IrO_X$-$TiO_2$-g-$C_3N_4$-Pt | 300W Xe lamp | 8.5 | 10 vol.% methanol | Not provided | 8 |
| $TiO_2$-NiS-Pt | 300W Xe lamp, AM I.5 filter | 4.7 | 20 vol.% methanol | 35 ℃ | 9 |
| $TiO_2$-Pt | LED light (λ=365 nm); 71 $mW/cm^2$ | 11.0 | 10 vol.% ethanol | Not provided | 10 |
| $TiO_2$-Pt | 300W Xe lamp | 10.0 | 10 vol.% methanol | 10 ℃ | 11 |

**Table S3.** Comparison of the activity of NMPs with other state-of-the-art $TiO_2$-noble metal

photocatalysts in hydrogen evolution.

| 20 nm Au-$TiO_2$ NMPs | Au/$TiO_2$ (wt%) | $TiO_2$ colloid (mL) | Au colloid (mL) | DCM (mL) | TBA $NO_3$ (mL) |
|---|---|---|---|---|---|
| | 10 | 1 | 3.5 | 5 | 0.5 |
| | 30 | 1 | 10.5 | 5 | 0.5 |
| | 50 | 1 | 17.5 | 5 | 0.5 |
| | 70 | 1 | 24.5 | 5 | 0.5 |
| | 90 | 1 | 31.5 | 5 | 0.5 |
| 30 nm Au-$TiO_2$ NMPs | Au/$TiO_2$ (wt%) | $TiO_2$ colloid (mL) | Au colloid (mL) | DCM (mL) | |
| | 10 | 1 | 2.2 | 5 | 0.5 |
| | 30 | 1 | 6.6 | 5 | 0.5 |
| | 50 | 1 | 11 | 5 | 0.5 |
| | 70 | 1 | 15.4 | 5 | 0.5 |
| | 90 | 1 | 19.8 | 5 | 0.5 |
| 40 nm Au-$TiO_2$ NMPs | Au/$TiO_2$ (wt%) | $TiO_2$ colloid (mL) | Au colloid (mL) | DCM (mL) | |
| | 10 | 1 | 1.5 | 5 | 0.5 |
| | 30 | 1 | 4.5 | 5 | 0.5 |
| | 50 | 1 | 7.5 | 5 | 0.5 |
| | 70 | 1 | 10.5 | 5 | 0.5 |
| | 90 | 1 | 13.5 | 5 | 0.5 |
| 20 nm Pt-$TiO_2$ | Pt/$TiO_2$ (wt%) | $TiO_2$ colloid (mL) | Pt colloid (mL) | DCM (mL) | |

| | | | | | |
|---|---|---|---|---|---|
| NMPs | | | | | |
| | 2.4 | 1 | 1 | 5 | 0.5 |
| | 7.2 | 1 | 3 | 5 | 0.5 |
| | 12.0 | 1 | 5 | 5 | 0.5 |
| | 16.8 | 1 | 7 | 5 | 0.5 |
| | 21.6 | 1 | 9 | 5 | 0.5 |
| 50 nm Pt-$TiO_2$ NMPs | Pt/$TiO_2$ (wt%) | $TiO_2$ colloid (mL) | Pt colloid (mL) | DCM (mL) | |
| | 2.4 | 1 | 1 | 5 | 0.5 |
| | 7.2 | 1 | 3 | 5 | 0.5 |
| | 12.0 | 1 | 5 | 5 | 0.5 |
| | 16.8 | 1 | 7 | 5 | 0.5 |
| | 21.6 | 1 | 9 | 5 | 0.5 |
| 75 nm Pt-$TiO_2$ NMPs | Pt/$TiO_2$ (wt%) | $TiO_2$ colloid (mL) | Pt colloid (mL) | DCM (mL) | |
| | 2.4 | 1 | 1 | 5 | 0.5 |
| | 7.2 | 1 | 3 | 5 | 0.5 |
| | 12.0 | 1 | 5 | 5 | 0.5 |
| | 16.8 | 1 | 7 | 5 | 0.5 |
| | 21.6 | 1 | 9 | 5 | 0.5 |
| Ag-$TiO_2$ NMPs | Ag/$TiO_2$ (wt%) | $TiO_2$ colloid (mL) | Ag colloid (mL) | DCM (mL) | TBA $NO_3$ (mL) |
| | 0.55% | 1 | 5 | 5 | 0.5 |
| $TiO_2$ NMPs | $TiO_2$ (wt%) | $TiO_2$ colloid | Metal colloid | DCM (mL) | TBA $NO_3$ |

| | | | | | |
|---|---|---|---|---|---|
| | | (mL) | | | (mL) |
| | 100 | 1 | 0 | 5 | 0.5 |

**Table S4.** The volume of all ingredients required for the fabrication of various types of metal-$TiO_2$ NMPs.

**Discussion S1. Calculation of the size of NMPs**

The size of NMPs can be calculated using the following equations:

The volume of one emulsion droplet ($V_e$) containing 10% PS can be expressed as:

$$V_e = \frac{4}{3}\pi(r_e)^3 \qquad (3)$$

where $r_e$ is the radius of the emulsion droplet.

The mass of this emulsion droplet can be expressed as:

$$m_e = \rho_e \times V_e = \rho_e \times \frac{4}{3}\pi(r_e)^3 \qquad (4)$$

where $\rho_e$ is the density of the emulsion droplet. Since one emulsion droplet only contains 10 wt.% PS, we assume that $\rho_e$ equals to the density of dichloromethane ($\rho_D$), which amounts to 1.33 g/cm$^3$.

After the complete evaporation of dichloromethane, the polystyrene precipitates out for the formation of NMPs. We then assume that the polystyrene interior of a single NMP is a perfect sphere, whose mass ($m_N$) can be expressed as:

$$m_N = \rho_N \times V_N = \rho_N \times \frac{4}{3}\pi(r_N)^3 \qquad (5)$$

where $\rho_N$ is the density of the NMPs, $V_N$ is the volume of the NMPs, and $r_N$ is the radius of the NMPs. Since NMPs are made up mostly of polystyrene, $\rho_N$ equals to the density of polystyrene at ~1 g/cm$^3$.

Since one emulsion droplet contains 10 wt.% PS, the mass of one NMP can also be expressed as:

$$m_N = 0.1 \times m_e \qquad (6)$$

$$\rho_N \times \frac{4}{3}\pi(r_N)^3 = 0.1 \times \rho_e \times \frac{4}{3}\pi(r_e)^3 \qquad (7)$$

$r_N$ can be calculated as:

$$r_N = \sqrt[3]{0.1 \times \frac{\rho_e}{\rho_N}} = \sqrt[3]{0.1 \times \frac{1.33}{1}} \approx 0.5 r_e \qquad (8)$$

Therefore, an initial emulsion droplet of ca. 300 um indeed results in the formation of NMP of ca. 150 µm. As a result, the surface area of one NMP is only 1/4 of that of its parent emulsion droplet.

**Discussion S2. Calculation of the weight percentage of $TiO_2$ in the Pt-$TiO_2$ NMPs:**

The amount of polystyrene used:

$$m_{polystyrene} = 0.1g/mL \times 5\ mL = 0.5\ g \quad (9)$$

The amount of $TiO_2$ used:

$$m_{TiO2} = 5\ mg/mL \times 1\ mL = 5\ mg \quad (10)$$

The theoretical percentage of $TiO_2$:

$$P_{TiO2} \approx \frac{5mg}{500mg} \approx 1\% \quad (11)$$

**Discussion S3. Calculation of $R_a$:**

Ra is an empirical value which was obtained based on the data obtained at optimum conditions for each particle size. For example, for 20 nm Pt nanoparticles, the particle density at optimum conditions was 190 particles $\mu m^{-2}$, and the harvested area equals to 0.90 of available area for hexagonally close packed circles. This gives the below equation:

$$\pi n_p\left(R_p + R_a\right)^2 = 0.9 \times 10^6 \quad (12)$$

where the particle radius ($R_p$) was 10 nm. Solving the above equation gives us $R_a \approx 28.8$ nm.

**Discussion S4. Calculation of $\theta$:**

The capture angle ($\theta$) reflects the ability of a metal nanoparticle to capture photogenerated electrons. It depends on the particle size and distance of the photogenerated electron from the metal nanoparticle and can be calculated using the following equation:

$$\sin\theta = \frac{R_p}{R_a + R_p} \quad (13)$$

$$\theta = \sin^{-1}\frac{R_p}{R_a + R_p} \quad (14)$$

REFERENCES

[1] Cao, S.; Piao, L. Considerations for a More Accurate Evaluation Method for Photocatalytic Water Splitting. *Angew. Chem. Int. Ed.* **2020**, *59*, 18312-18320.

[2] Gao, D.; Deng, P.; Zhang, J.; Zhang, L.; Wang, X.; Yu, H.; Yu, J. Reversing Free-Electron Transfer of $MoS_{2+x}$ Cocatalyst for Optimizing Antibonding-Orbital Occupancy Enables High Photocatalytic H2 Evolution. *Angew. Chem. Int. Ed.* **2023**, *62*, e202304559.

[3] Gao, D.; Xu, J.; Wang, L.; Zhu, B.; Yu, H.; Yu, J. Optimizing Atomic Hydrogen Desorption of Sulfur-Rich $NiS_{1+x}$ Cocatalyst for Boosting Photocatalytic $H_2$ Evolution. *Adv. Mater.* **2022**, *34*, 2108475.

[4] Karimi Estahbanati, M. R.; Feilizadeh, M.; Iliuta, M. C. Photocatalytic Valorization of Glycerol to Hydrogen: Optimization of Operating Parameters by Artificial Neural Network. *Appl. Catal. B* **2017**, *209*, 483-492.

[5] Jung, M.; Hart, J. N.; Boensch, D.; Scott, J.; Ng, Y. H.; Amal, R. Hydrogen Evolution via Glycerol Photoreforming Over Cu–Pt Nanoalloys on $TiO_2$. *Appl. Catal. B* **2016**, *518*, 221-230.

[6] Wang, Z.; Yang, Q., Liu, W.; Ran, H.; Zhang, C.; Han, X.; He, X.; Wang, X.; Hu, G. Optical Porous Hollow-Boxes Assembled by $SrSO_4$/$TiO_2$/Pt Nanoparticles for High Performance of Photocatalytic $H_2$ Evolution. *Nano Energy* **2019**, *59*, 129-137.

[7] Hu, X.; Song, J.; Luo, J.; Zhang, H.; Sun, Z.; Li, C.; Zheng, S.; Liu, Q. Single-Atomic Pt Sites Anchored on Defective $TiO_2$ Nanosheets as a Superior Photocatalyst for Hydrogen Evolution. *J. Energy Chem.* **2021**, *62*, 1-10.

[8] Moon, H. S.; Hsiao, K. C.; Wu, M. C.; Yun, Y.; Hsu, Y. J.; Yong, K. Spatial Separation of Cocatalysts on Z-Scheme Organic/Inorganic Heterostructure Hollow Spheres for Enhanced Photocatalytic $H_2$ Evolution and In-Depth Analysis of the Charge-Transfer Mechanism. *Adv. Mater.* **2023**, *35*, 2200172.

[9] Wang, S.; Sun, H.; Qiao, P.; Li, Z.; Xie, Y.; Zhou, W. NiS/Pt Nanoparticles Co-Decorated Black Mesoporous $TiO_2$ Hollow Nanotube Assemblies as Efficient Hydrogen Evolution Photocatalysts. *Appl. Mater. Today* **2021**, *22*, 100977.

[10] Vasilchenko, D.; Topchiyan, P.; Tsygankova, A.; Asanova, T.; Kolesov, B.; Bukhtiyarov, A.; Kurenkova, A.; Kozlovam E. Plasmon Induced Nano Au Particle Decorated over S, N-Modified $TiO_2$ for Exceptional Photocatalytic Hydrogen Evolution under Visible Light. *ACS Appl. Mater. Interfaces* **2020**, *12*, 48631-48641.

[11] He, Y.; Li, P.; Liu, W.; An, L.; Qu, D.; Wang, X.; Sun, Z. The Critical Factors of Photocatalytic $H_2$ Production from Seawater by Using $TiO_2$ as Photocatalyst. *Nano Res.* **2023**, *16*, 4620-4624.
